# Labial Necrotizing Fasciitis Caused by Pelvic Eggerthia catenaformis Infection

**DOI:** 10.7759/cureus.53625

**Published:** 2024-02-05

**Authors:** Felix Yang, Mira Malavia, Ashna Chali, Jared Keeler

**Affiliations:** 1 Department of Internal Medicine, University of Missouri Kansas City School of Medicine, Kansas City, USA

**Keywords:** perioperative outcomes, cellulitis, abscess, infection, surgical debridement, necrotizing fasciitis, case report

## Abstract

A patient with comorbid diabetes mellitus, obesity, and hypertension acutely presented to the ED due to labial cellulitis with rapidly progressing symptoms of systemic inflammation. Clinical examination revealed fever and groin pain that was tender to palpation. Initial contrast-enhanced CT scans showed labial cellulitis extending to the inguinal canal, with later CT imaging findings of subcutaneous air indicative of necrotizing fasciitis (NF). Antimicrobial therapy was initiated empirically and later tailored to culture antibiogram. The patient underwent acute surgical abscess drainage and tissue debridement but was transferred to the surgical intensive care unit (SICU) due to postoperative blood loss and hypotension. Two additional surgical procedures were needed before sufficient drainage was achieved, and *Eggerthia catenaformis* (*E. catenaformis*) was isolated from all samples. Due to the extent of the infection, the patient was admitted for a total of 16 days, with five days spent in the SICU. They recovered completely due to adequate surgery and antimicrobial therapy for a total of 24 days. Here, we present the third reported case of NF due to *E. catenaformis*while emphasizing timely treatment with empiric antibiotics and surgical intervention.

## Introduction

*Eggerthia catenaformis* (*E. catenaformis*) is an anaerobic, non-spore-forming, gram-positive bacillus that is found in the human fecal microbiome. It was first reported by Eggerth (1935), who discovered the species after isolating several colonies from fecal samples on blood agar [1]. Since its discovery, *E. catenaformis* has been associated with a multitude of infections, including descending necrotizing mediastinitis, peritonitis, pulmonary infection, and dental abscess [2-7].

Necrotizing fasciitis (NF) is a bacterial soft tissue infection that progresses rapidly and can result in widespread tissue damage, mostly to the subcutaneous tissue and deep fascia. If not addressed, it can lead to sepsis, organ failure, and death [8]. There are two major classifications: polymicrobial (type one) and monomicrobial (type two). Polymicrobial NF is more common and is caused by both anaerobic and aerobic bacteria, which leads to gaseous infiltration of tissue resembling gas gangrene. Monomicrobial NF is commonly caused by gram-positive organisms, such as group A streptococci [9]. Other organisms that have been found in cases of NF include *Enterococci*, *Bacteroides*, *Pseudomonas*, *Klebsiella*, *Clostridium*, *Aeromonas*, and *Vibrio vulnificus* [9-11]. This condition usually results after an initial injury to the skin, such as surgical wounds, lacerations, animal bites, or scratches. Typically, this infection quickly transits into the fascial layer of tissue and can rapidly spread to cause liquefactive necrosis at all tissue levels. Due to this pathophysiology, it is not uncommon to see delayed surface skin findings until a more severe disease progresses. At this stage, common symptoms of the infection include out-of-proportion pain, warmth, erythema, discoloration, tenderness, skin sloughing, and bullae formation. Advanced symptoms of NF include pyrexia, tachycardia, and sepsis [11].

NF is primarily a clinical diagnosis that incorporates physical exam findings, laboratory results, imaging, and surgical exploration. The characteristic appearance is dull, gray fascia with stringy areas of necrosis and brown exudate, with no true pus detected. The causative bacteria can be determined from the culture and gram stain of specimens collected from deep tissue or blood culture [10]. It is important to note that lab values by themselves are not sufficient to make a diagnosis of NF. The laboratory risk indicator for NF (LRINEC) was developed in 2004 to screen for NF. However, later studies found a high false-positive rate in patients with confirmed cases of cellulitis, low sensitivity among confirmed cases of NF, and even a case of a patient who had NF with an LRINEC score of zero [12,13]. Among patients with a confirmed diagnosis of NF, a retrospective analysis by Neeki et al. demonstrated a statistically significant proportion of patients misclassified as being "low-risk" for NF (p<0.0001) [12]. This indicates that lab values alone may not be an accurate predictor and may lead to a high proportion of delayed and inappropriate medical management. Imaging can also be used to aid in the diagnosis of NF, as CT and MRI scans often show edema extending along the fascial plane. However, these findings may not be apparent in the early stages, and treatment should not be delayed to obtain imaging evidence when there is high clinical suspicion for NF [10,14].

To our knowledge, there have only been two other cases in the literature of NF involving *E. catenaformis *[15,16]. We present an unusual case of labial infection with *E. catenaformis, *resulting in NF in a patient with comorbid diabetes, hypertension, obesity, and renal dysfunction. This is the third case of pelvic infection with *E. catenaformis* described in the literature.

## Case presentation

**Figure 1:**
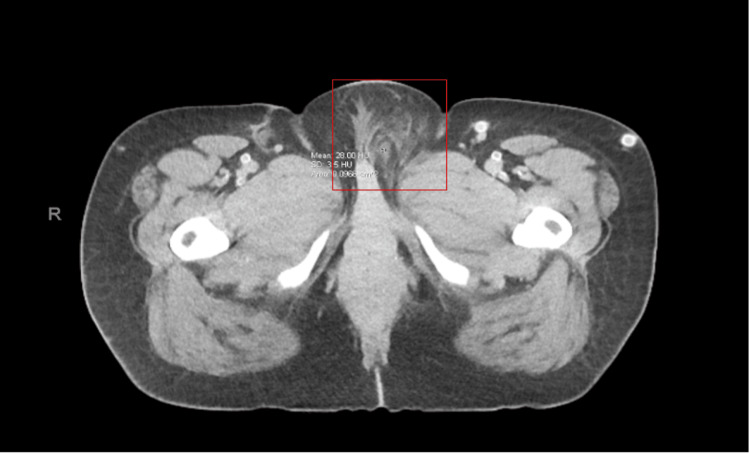
Contrast CT of the abdomen and pelvis on the day of admission Left labial cellulitis (red box) with no soft tissue gas or abscess collections

A woman in her 40s with a past medical history of diabetes mellitus type two, end-stage renal disease on hemodialysis, obesity (BMI 43 kg/m2), hypertension, and obstructive sleep apnea was suspected of having NF. The patient first presented to the ED following two days of groin pain in the vaginal area with associated tenderness to palpation and subjective fever. She denied any vaginal discharge, unusual bleeding, dysuria, hematuria, nausea, vomiting, or abdominal pain. On physical examination, left external labia and mons pubis swelling and erythema were noted with moderate induration of the labia. No fluctuance, crepitus, or significant drainage was discovered on the exam. The patient was awake with a temperature of 100.3°F, a heart rate of 115 BPM, and a blood pressure of 134/61 mmHg. Laboratory work showed elevated WBC, elevated CRP, and creatinine (9.86) along with other significant lab abnormalities (Table 1), and a CT abdomen/pelvis with contrast found left labia cellulitis with inflammatory and phlegmonous changes extending into the left inguinal canal (Figure 1). Pain in the ED was managed with Toradol and Tylenol, and a dose of clindamycin was administered. She was then admitted to inpatient medicine due to concerns about the potential infection of her left femoral arteriovenous dialysis (AV) graft and potential complications from her past medical history.

**Table 1:** Laboratory investigation results of the patient on the day of admission MCV: mean corpuscular volume, MCHC: mean corpuscular hemoglobin concentration

Parameter	Results	Reference range
White blood cells (× 10^9^/L)	18.10	4.5-11.0
Red blood cells (× 10^12^/L)	3.59	4.3-5.9
Hemoglobin (g/dL)	9.8	13.5-17.5
Hematocrit (%)	30.3	41-53
MCV (fl)	84.4	80-100
MCHC (g/dL)	32.4	31%-36
Platelets (× 10^9^/L)	210	150-450
Neutrophils (%)	85	2.5-8.0
Lymphocytes (%)	7	1.0-4.0
Monocytes (%)	6	0.1-0.7
Eosinophils (%)	0	0.05-0.50
Basophils (%)	1	0.025-0.100
C-reactive protein (mg/L)	292	<10.0
Procalcitonin (ng/mL)	9.2	<0.1

**Figure 2:**
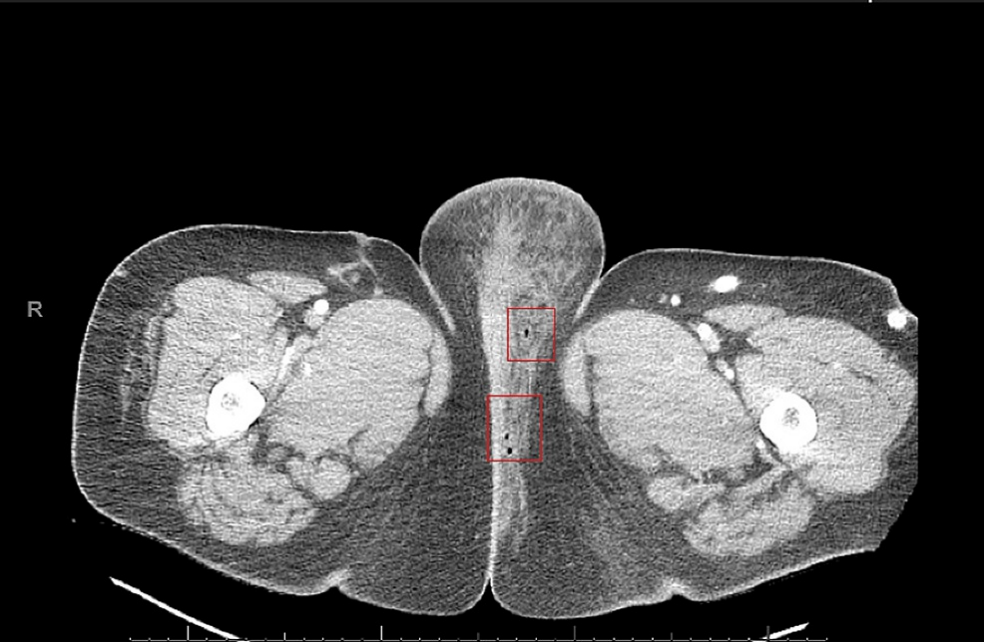
Contrast CT of the pelvis confirming disease progression Worsening left labial cellulitis with interval development of subcutaneous air (red boxes) concerning for an aggressive soft tissue infection

During her hospital stay, the patient experienced worsening leukocytosis (WBC 25.30), rising CRP (CRP 381.9), and increasing pain. Repeated fevers spiking up to 100.4°F and lingering erythema prompted a repeat CT scan due to concerns of a potential abscess. This scan showed interval development of subcutaneous air, which was concerning for an aggressive soft tissue infection (Figure 2). Blood cultures found no growth, but wound and groin abscess cultures were positive for *E. catenaformis*, as well as *Gleimia* and *Ergethella *species. With these findings, an infectious diseases consult was placed, and concern for NF prompted empiric, broad-spectrum antibiotic treatment. The patient was scheduled for 10 days of piperacillin-tazobactam and vancomycin and five days of clindamycin. Antibiotic susceptibility testing was not performed. Their home diabetes management was an insulin pump with no additional oral or parenteral medications. She reported having run out of insulin one hour before arriving in the ED. In the hospital, a basal-bolus insulin regimen of insulin glargine 40U and lispro 10U with meals was started and later increased to 45U and 12U, respectively. Overnight blood glucose was fairly well controlled (175-219 mg/dL) via continuous glucose monitoring. The only reported hemoglobin A1c was 6.8% and was measured four days after her presentation to the ED.

**Figure 3:**
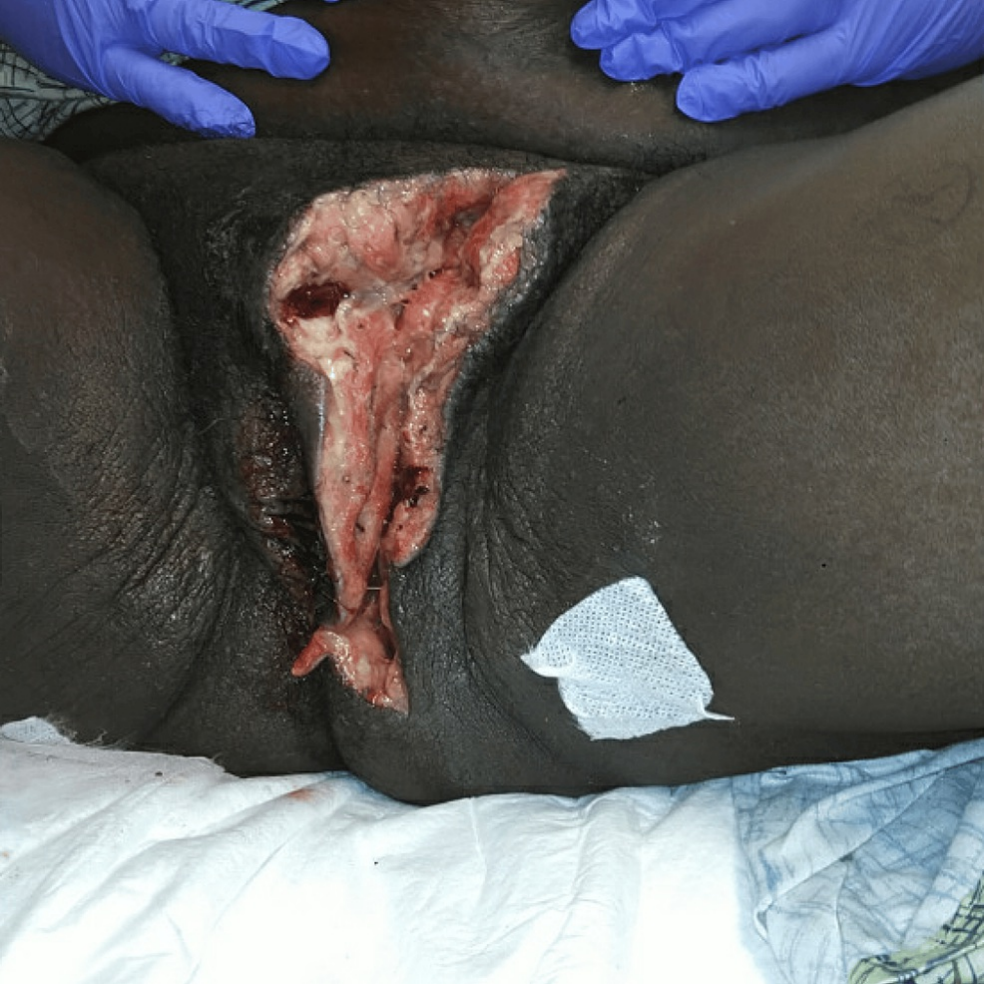
Surgical wound after final debridement and drainage 15.5 cm x 6 cm x 3 cm left vulvar surgical wound residual serosanguineous drainage. Wound edges were moist pink with adipose tissue noted within the wound bed

During her hospital stay, the patient was evaluated by both OB/GYN and general surgery due to worsening swelling, up-trending leukocytosis, and pain. The general surgery team recommended urgent irrigation and debridement after evaluating the patient due to concern about a necrotizing soft tissue infection. Four days after the patient’s initial presentation in the ED, she was taken to the operating room for incision and drainage due to increased swelling and clinical deterioration, which were all concerning for necrotizing soft tissue infection. Incision and drainage with concurrent debridement were performed. Dissection through the subcutaneous tissue discovered a pocket of gray “dishwater-like” fluid with diseased fat in the superior and inferior parts of the labia. The wound was then packed with Betadine-soaked Kerlix and covered with an APD pad. Overall, the procedure was uncomplicated, and the patient was transferred to the post-anesthesia care unit (PACU) (Figure 3).

**Figure 4:**
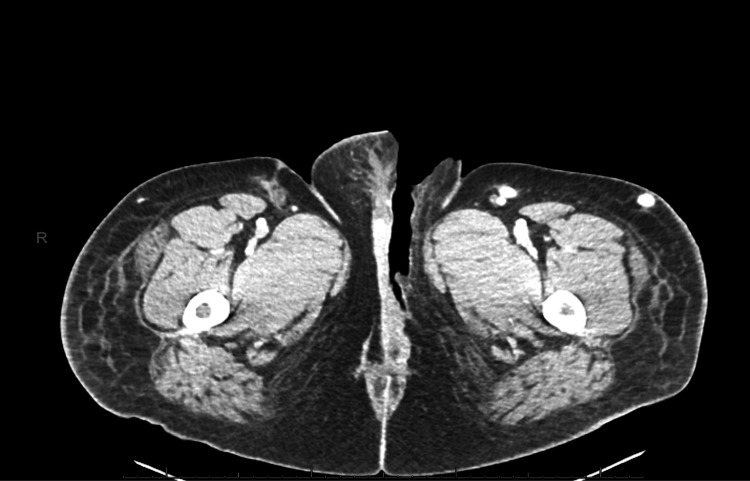
Post-surgical contrast CT of the pelvis Post-surgical changes of the labial tissue with no obvious aggressive new cross-fascial inflammatory extension or drainable fluid collection

While in the PACU, the patient was noted to have superficial dermal oozing from the surgical site, was getting deep vein thrombosis prophylaxis, and was also noted to be hypotensive with mean arterial pressures measuring in the 50s. Hemoglobin at this time was 6.3, which necessitated transfusion, and initial resuscitation was performed with 2 L of normal saline and two units of packed red blood cells (pRBCs). However, blood pressure remained low, and further resuscitation was unable to be performed due to difficult IV access. It was determined that the patient would require ICU status due to the maximum capacity in the progressive care unit, and she was subsequently upgraded to the surgical intensive care unit (SICU). Repeat debridement, drainage, and washout was performed an additional two times after SICU admission. A follow-up CT abdomen/pelvis was performed two days after SICU admission, and no further signs of disease progression were evident (Figure 4). Hemoglobin remained stable after a total of five units of pRBCs, and the patient was transferred back to the medicine service.

The patient was also seen by wound care two times after her final incision and drainage. Daily dressing changes with lightly packed Vashe-soaked gauze, covered with ABD pads, and secured with mesh underwear were recommended. The peri-wound was also treated with Cavilon Advanced Skin Protectant, and Prisma collagen was placed in the wound bed. Leukocytosis down-trended daily, and overall clinical status improved. They were discharged 16 days after the initial presentation with IV ertapenem and vancomycin and the patient’s scheduled dialysis for an additional 14 days. It was noted that the wound was healing well with minimal brown drainage, closure of most of the incision, and minimal pain at both inpatient and general surgery follow-up visits. On observation, there was no erythema, fluctuance, or purulence from the wound. One month after hospital discharge, the patient was seen by wound care again, who noted a pale pink and dry incisional area with surrounding scarring that was free of inflammation. Pain improved throughout treatment, and the patient denied any lingering symptoms.

## Discussion

We report the third case in the literature of NF due to *E. catenaformis*. NF is a rapidly evolving soft-tissue infection characterized by spread along the fascial planes with fascial ischemia. Based on our case, we theorize that the most probable routes for microbial infection include fecal contamination and odontogenic transmission. However, several other routes of invasion have been described, including blunt and penetrating trauma, postoperative complications, insect bites, and idiopathic etiology [9,11]. There is a limited amount of literature describing cases of *E. catenaformis* and its pathophysiology in causing infection. However, it seems that odontogenic infection has the most supported relationship to necrotizing infections that have the potential to spread to various locations in the body [2-6,15]. Hematogenous spread from dental infections, as proposed by Kordjian is also a possible risk factor for soft tissue infection development, although our patient was not screened for any dental infection foci [17]. Cases of deep tissue infection from *E. catenaformis* have also been documented in the absence of dental disease [18]. Rahman et al. additionally identified two genes related to the virulence and resistance of *E. catenaformis *to tetracycline antibiotics. They summarized that oral pathogenic *E. catenaformis* has the potential to cause infection without dental disease, although infection severity is exacerbated if mucosal lesions are present [18]. While the patient’s AV graft was initially a concern due to the risk of local site infection and bacteremia, repeated blood cultures drawn throughout her hospital course were all negative. Thus, we concluded that the hematogenous spread of infection was not likely in this patient.

Excluding our report, we identified eight total clinical cases of *E. catenaformis* infections so far in the literature with most of them being odontogenic infections with subsequent abscess formation. Early diagnosis and immediate therapeutic measures such as IV broad-spectrum antibiotics and debridement are key to increasing the odds of survival, and the time to surgical intervention was shown to be an important predictor of survival [15]. While there is no gold standard in regards to the ideal length of time to intervention, Infectious Diseases Society of America guidelines for polymicrobial necrotizing infections recommend surgical debridement along with empiric vancomycin or linezolid in combination with piperacillin-tazobactam, a carbapenem, or ceftriaxone-metronidazole [8,9]. Tailoring of antibiotics should follow based on local antibiograms [9]. Frequent monitoring of wound margins should be performed to notice disease progression early on and allow quick surgical source control [8,14].

While imaging should not delay treatment, our patient’s CT imaging depicting a worsening necrotizing infection prompted immediate incision and drainage in addition to antimicrobial pharmacotherapy. Kordjian et al. described the first case of *E. catenaformis* bacteremia in a patient with dental abscess and cervical fluid accumulation treated with antibiotic therapy and surgical interventions [17]. In their report, minimal inhibitory concentration (MIC) analysis showed that the *E. catenaformis* strain was multisensitive with a low MIC to penicillin, clindamycin, metronidazole, meropenem, moxifloxacin, and vancomycin. In the clinical case of Duport et al., the isolated *E. catenaformis* strain was multisensitive but had a moderately high MIC value for metronidazole [4]. Multi-drug sensitivity was also shown in both reports of pelvic *E. catenaformis* infections, although resistance to metronidazole and moxifloxacin was shown to be possible. In both cases, a similar management approach was taken, which involved the initiation of a beta-lactamase antibiotic, metronidazole, and clindamycin with the addition of vancomycin by Illg et al. [15,16].

Our patient also had underlying medical conditions that are associated with an increased risk of developing soft tissue and polymicrobial infections [9]. Certain exogenic habits, such as smoking, could also negatively affect the immune system and impair wound healing. While it was documented that our patient was a never-smoker, her comorbid diabetes, severe obesity, and end-stage renal disease potentially led to a vulnerable immune system susceptible to developing NF [5]. Both diabetes and obesity are known risk factors for NF and have also been demonstrated to be the most prevalent among patients with pelvic NF [19]. With impaired wound healing and increased susceptibility to infection, diabetes itself increases the risk of developing such a kind of systemic infection [20]. It can also potentially be a major contributing factor to mortality, despite appropriate interventions [16]. Initiation and maintenance of a diabetes treatment early on in treatment, as seen in both the report by Illg et al. and our own, may prove to improve patient outcomes and decrease mortality [15]. Fortunately for our patient, the infection was adequately controlled after surgery and pharmacotherapy, with no further complications in follow-up appointments.

## Conclusions

*E. catenaformis* may cause severe infections in a variety of locations in the body, although it has been mostly described as causing dental abscesses. We report the third case of pelvic NF caused by *E. catenaformis* with an undetermined etiology at the time of hospital discharge. While not applicable to our case, odontogenic infection and hematogenous spread from a primary source have been demonstrated in the literature to be risk factors for pelvic NF from this organism. The time from disease development to pharmacotherapy and surgical interventions is important for improved patient outcomes. We also highlight the importance of combined treatment with broad-spectrum IV antibiotics and surgery, as that has been demonstrated to be effective in maximizing survivability from this infection. Although reported strains of *E. catenaformis* seem to be multisensitive to several antibiotics; physicians should initiate timely treatment with both antibiotics and surgery, especially in patients with comorbid, chronic diseases.

## References

[1] Eggerth AH (1935). The Gram-positive non-spore-bearing anaerobic bacilli of human feces. J Bacteriol.

[2] Graziani A, Tamburini MV, Congestrì F, Graziani L, Sama MG, Caroli G, Spaggiari R (2023). Descending necrotizing mediastinitis caused by retro-pharyngeal Eggerthia catenaformis infection. Germs.

[3] Özbay BO, Bastuğ A, Köksal Cevher Ş, Yenigün EC, Mumcuoğlu İ, Bodur H (2022). Eggerthia catenaformis-related peritonitis in a patient with peritoneal dialysis. Anaerobe.

[4] Duport P, Miltgen G, Kebbabi C, Belmonte O, Coolen-Allou N, Allyn J, Allou N (2018). First case of pleural empyema and pulmonary abscess caused by Eggerthia catenaformis. Anaerobe.

[5] Sakkas A, Nolte I, Heil S, Mayer B, Kargus S, Mischkowski RA, Thiele OC (2021). Eggerthia catenaformis infection originating from a dental abscess causes severe intestinal complications and osteomyelitis of the jaw. GMS Interdiscip Plast Reconstr Surg DGPW.

[6] Krarup JF, Nielsen HL, Danstrup CS (2021). Severe deep neck space infection caused by Eggerthia catenaformis. BMJ Case Rep.

[7] Akashi M, Tanaka K, Kusumoto J, Furudoi S, Hosoda K, Komori T (2017). Brain abscess potentially resulting from odontogenic focus: report of three cases and a literature review. J Maxillofac Oral Surg.

[8] Chen LL, Fasolka B, Treacy C (2020). Necrotizing fasciitis: a comprehensive review. Nursing.

[9] Stevens DL, Bryant AE (2017). Necrotizing soft-tissue infections. N Engl J Med.

[10] Stevens DL, Bisno AL, Chambers HF (2014). Practice guidelines for the diagnosis and management of skin and soft tissue infections: 2014 update by the Infectious Diseases Society of America. Clin Infect Dis.

[11] Salati SA (2022). Necrotizing fasciitis a review. Pol Przegl Chir.

[12] Neeki MM, Dong F, Au C (2017). Evaluating the laboratory risk indicator to differentiate cellulitis from necrotizing fasciitis in the emergency department. West J Emerg Med.

[13] Wilson MP, Schneir AB (2013). A case of necrotizing fasciitis with a LRINEC score of zero: clinical suspicion should trump scoring systems. J Emerg Med.

[14] Sartelli M, Guirao X, Hardcastle TC (2018). 2018 WSES/SIS-E consensus conference: recommendations for the management of skin and soft-tissue infections. World J Emerg Surg.

[15] Illg C, Kolbenschlag J, Schäfer RC, Daigeler A, Krauss S (2022). First report of polymicrobial necrotizing fasciitis caused by Eggerthia catenaformis and Finegoldia magna. World J Emerg Med.

[16] Wellkamp L, Pfannkuchen B, Schilawa D (2020). First clinical case of extensive necrotizing fasciitis of the abdominal wall and groin area with fatal outcome caused by Eggerthia catenaformis. Clin Surg.

[17] Kordjian HH, Schultz JD, Rosenvinge FS, Møller J, Pedersen RM (2015). First clinical description of Eggerthia catenaformis bacteremia in a patient with dental abscess. Anaerobe.

[18] Rahman MA, Mullany P, Roberts AP (2017). Draft genome sequence of Eggerthia catenaformis strain MAR1 isolated from saliva of healthy humans. Genome Announc.

[19] Nakayama J, Busse R (2010). An analysis of vulvar necrotizing fasciitis in the unique and ethnically diverse Hawaiian population. Hawaii Med J.

[20] Giaccari A, Sorice G, Muscogiuri G (2009). Glucose toxicity: the leading actor in the pathogenesis and clinical history of type 2 diabetes - mechanisms and potentials for treatment. Nutr Metab Cardiovasc Dis.

